# PDIA6 contributes to aerobic glycolysis and cancer progression in oral squamous cell carcinoma

**DOI:** 10.1186/s12957-021-02190-w

**Published:** 2021-03-24

**Authors:** Ling Mao, Xiaoweng Wu, Zhengpeng Gong, Ming Yu, Zhi Huang

**Affiliations:** 1 The Laboratory of Head and Neck Cancer Research, Hospital and School of Stomatology, Guizhou Medical University, Guiyang, 550004 People’s Republic of China; 2 Imaging department, Liaocheng People’s Hospital, Liaocheng, China; 3 Department of Imaging, Liaocheng People’s Hospital, 252000 Guiyang, People’s Republic of China; 4 Department of Otorhinolaryngology, The Affiliated Hospital of Guizhou Medical University, Guiyang, China; 5 Laryngology and Otology, the Affiliated Baiyun Hospital of Guizhou Medical University, No. 108 Gangyu Road, Guiyang, 550005 People’s Republic of China; 6 Department of Interventional Radiology, the Affiliated Cancer Hospital of Guizhou Medical University, Guiyang, 550002 People’s Republic of China; 7 College of Basic Medicine, Guizhou Medical University, No. 1 Beijingxi Road, Guiyang, 550002 People’s Republic of China

**Keywords:** PDIA6, Proliferation, Migration, Aerobic glycolysis, Tumorigenesis

## Abstract

**Background/objective:**

Accumulated evidence has demonstrated that aerobic glycolysis serves as a regulator of tumor cell growth, invasion, and angiogenesis. Herein, we explored the role of protein disulfide isomerase family 6 (PDIA6) in the aerobic glycolysis and the progression of oral squamous cell carcinoma (OSCC).

**Methods:**

The expression pattern of PDIA6 in OSCC tissues was determined by qPCR and western blotting. Lentivirus and small interfering RNAs (siRNAs) were introduced into cells to upregulate and downregulate PDIA6 expression. CCK-8, flow cytometry, transwell, and xenotransplantation models were applied to detect cell proliferation, apoptosis, migration, invasion, and tumorigenesis, respectively.

**Results:**

A high expression pattern of PDIA6 was observed in OSCC tissues, which was closely associated with lower overall survival and malignant clinical features in OSCC. Compared with the control group, overexpression of PDIA6 induced significant enhancements in cell growth, migration, invasiveness, and tumorigenesis and decreased cell apoptosis, while knockdown of PDIA6 caused opposite results. In addition, overexpression of PDIA6 increased glucose consumption, lactate production, and ATP level in OSCC cells.

**Conclusion:**

This study demonstrated that PDIA6 expression was elevated in OSCC tissues, and overexpression of it promoted aerobic glycolysis and OSCC progression.

## Introduction

Oral squamous cell carcinoma (OSCC), as a major type of head and neck cancer, ranks the sixth most common malignant tumor in the world and ranks eighth in cancer-related mortalities [1]. It is reported that about 354,800 new cases are diagnosed with OSCC and 177,400 people died of OSCC annually [2]. Despite big progresses being achieved in treatment and diagnosis, the 5-year survival rate for OSCC is only about 50% and the regional recurrence rate is 33~40% [3, 4]. High invasiveness and metastasis rates are two main reasons for the worse outcome of OSCC [5]. Thus, it is urgent to uncover the molecular mechanisms underlying OSCC development.

Aerobic glycolysis, also known as the Warburg effect, is one of the main features of cancer cells [6, 7]. Generally, glucose uptake and lactate production are increased in tumor cells via the aerobic glycolysis pathway to meet the requirement of elevated bioenergetic and biosynthetic demand for growth, metastasis, and invasion, leading to lactate content increase and pH value decrease [8]. Evidence has demonstrated that elevated lactate level is closely associated with tumor growth, invasion, and angiogenesis [9, 10]. For example, Cai et al. [11] reported that lactate dehydrogenase A (LDHA) was highly expressed in OSCC tissues and cell lines, and knockdown of it repressed cell proliferation, migration, invasion, and in vivo tumor formation through inhibiting glycolysis. Targeting aerobic glycolysis pathway is a promising method for cancer treatment [12].

Protein disulfide isomerase family 6 (PDIA6), also known as ERP5 or P5, belongs to the protein disulfide isomerase (PDI) family that assists protein folding and inhibits the polymerization of unfolded substrates and oxidoreductases to facilitate the formation of disulfide bonds [13, 14]. Recently, researches have demonstrated that PDIA6 is overexpressed in several kinds of human cancers and serves as an oncogene, such as lung cancer [15], bladder cancer [16], and hepatocellular carcinoma [17]. However, whether PDIA6 is involved in the aerobic glycolysis and the progression of OSCC still needs to be elucidated.

In the present study, we aimed to explore the expression pattern of PDIA6 in OSCC tissues and to reveal its role in the aerobic glycolysis and the progression of OSCC.

## Materials and methods

### Ethics statement

The current study has got the approval of the ethics committee of Guizhou Medical University (No. 2001221).

### Clinical tissue samples

Fifty-eight OSCC tissues and the adjacent noncancerous tissues were obtained from patients with primary OSCC undergoing surgical resection. No patients received chemotherapy or radiotherapy prior to surgery. All specimens were confirmed by two pathologists. The written informed consents were signed by every patient, and the current study has got the approval of the ethics committee of Guizhou Medical University. All tissue samples were rapidly placed at − 80 °C for further study. The OSCC patients were divided into PDIA6 high expression and low expression groups based on the mRNA levels of PDIA6. In detail, the 2^−∆∆Ct^ method was used to calculate the relative expression level of PDIA6 mRNA to the adjacent noncancerous tissues in every cancer tissue sample. Then, the OSCC patients were divided into the high expression group and low expression group according to the relative expression median. If the expression level of PDIA6 was ≥ the median (which was 1.9 in the current study), PDIA6 was considered as high expression. If the expression level of PDIA6 was < the median, PDIA6 was considered as low expression.

### Cell lines and culture method

Two human OSCC cell lines, SCC9 and Cal27, were all purchased from ATCC (Manassas, VA, USA). SCC9 cells were seeded in Eagle’s medium and Ham’s F12 medium, and Cal27 cells were placed in DMEM, with 10% fetal bovine serum (FBS) and 1% (v/v) penicillin/streptomycin. All cells were kept in a humidified atmosphere with 5% CO_2_ with a stationary temperature of 37 °C. Cell culture medium and FBS were purchased from Invitrogen (Carlsbad, CA, USA).

### Lentivirus and small interfering RNAs (siRNAs)

The lentivirus vectors applied to overexpress PDIA6 (called OE-PDIA6), and the siRNA used to downregulate PDIA6 (called si-PDIA6) and their negative controls (OE-NC, si-NC) were all purchased from Shanghai GenePharma Co., LTD (Shanghai, China). The lentivirus vectors were introduced into cells via cell infection by using polybrene, and si-PDIA6 and si-NC were introduced into cells via cell transfection using Lipofectamine 2000 reagent (Invitrogen). The infected cells were incubated in G418 (100 μg/ml) for 14 days to establish the stable cell lines which were used in the in vivo assay.

### Real-time quantitative polymerase chain reaction (qPCR)

Total RNA was extracted from tissues and cells by using TRIzol reagent (Life Technologies, Waltham, MA, USA) referring to the manufacturer’s manual. Then, the cDNA was produced using a PrimeScriptTM 1st Strand cDNA Synthesis Kit (Takara, Dalian, China). After that, the cDNA was served as substrate for qPCR with SYBR Premix Ex Taq (Takara) on an ABI 7900 system (Applied Biosystems, Foster City, CA, USA). The 2^−ΔΔCt^ method was applied to analyze the relative levels of mRNAs after being normalized to that of the expression level of β-ACTIN. The sequences used in this experiment are listed in Table 1.

**Table 1:** Primer sequences

Gene	Sense (5′-3′)	Antisense (5′-3′)
PDIA6	CACTAGGCGCTCACTGTTC	GAGGGATCTCGCTCCTGGAA
β-actin	TGCGTGACATTAAGGAGA AG	GCTCGTAGCTCTTCTCCA

### Western blotting assay

Total protein was obtained from cells using the RIPA lysis buffer (Beyotime, Jiangsu, China), supplemented with 1% (v/v) protease inhibitors (Beyotime). Then, 30 μg protein sample obtained from every group was separated by 10% sodium dodecyl sulfate-polyacrylamide gel electrophoresis, followed by transformation into the polyvinylidene difluoride membranes (Millipore, Billerica, MA, USA). The membranes were then successively incubated with 5% fat-free milk for 1 h at room temperature and probed with the primary antibodies, including PDIA6 (1:2,000 dilution; No. ab11432, Abcam, Cambridge, MA, USA) and β-actin (No. ab8226, Abcam, 1:5,000 dilution) overnight at 4 °C. After that, the membranes were probed with secondary antibodies (Abcam) for 1 h. Protein signals were detected using iBright CL750 (Thermo Fisher Scientific, MA, USA) after incubation with ECL (Thermo Fisher Scientific).

### CCK-8 assay

The proliferation assay was performed with the Cell Counting Kit-8 (CCK-8) assay. First, the OSCC cells (2500 cells for each well) were seeded in 96-well plates, allowed to adhere, transfected, and then cultured at 37 °C for 0, 24, 48, and 72 h. Then, 10 μl CCK-8 solution (Abcam) was added to each well and allowed to incubate for further 4 h at 37 °C. The OD values at 450 nm were determined using a spectrophotometer (BioTek Instruments, Winooski, VT, USA).

### Flow cytometry assay

Cell apoptotic rates were determined by using the flow cytometry assay. The cells were resuspended in 500 μl 1X binding buffer and incubated with 5 μl Annexin V-FITC in the dark for 15 min, followed by incubation with propidium iodide (PI) in the dark for 5 min. The cells were then tested for apoptosis on flow cytometry (BD Biosciences, Franklin Lakes, NJ, USA). The results were analyzed using Flowjo 7.6 software.

### Transwell chamber assay

Cell migration and invasiveness were assessed with Transwell chambers (8 μm, Corning, NY, USA) in 24-well plates using uncoated or Matrigel (BD Biosciences, San Jose, USA)-coated membranes. In brief, OSCC cells in the culture medium with 1% FBS were seeded in the upper well with 1 × 10^5^ cells for each well, and 600 μl cell culture medium with 10% FBS was added in the lower chambers. After 24 h or 48 h of incubation, the cells that did not penetrate the membrane on the upper chamber were removed with a cotton swab. Cells in the lower membrane were fixed with methanol and stained with 0.1% crystal violet solution. After washing with PBS for 4–5 times, the numbers of stained cells in 6 random fields were counted under an inverted microscope.

### Detection of lactate production, glucose consumption, and ATP levels

OSCC cells were inoculated in 6-well plates at a density of 2 × 10^5^ cells and allowed to attach. Following 24 h of incubation, the medium was collected to measure lactate production and glucose consumption with commercial kits from Nanjing Jiancheng Biotech (Jiangsu, China; A019) and Applygen Co., LTD (Beijing, China; No. E1010) according to the manufacturer’s descriptions, respectively. ATP levels were tested using an ATP Colorimetric/Fluorometric Assay Kit (Sigma-Aldrich, MO, USA) according to the manufacturer’s instructions.

### Tumor xenograft

Male NOD/SCID mice aged 5–6 weeks obtained from Wuhan Huaguenke Biotechnology Co. LTD (Hubei, China) were randomly divided into OE-NC and OE-PDIA6 groups (*n* = 5 for each group). This animal assay has got the approval of the Experiment Ethics Committee of Guizhou Medical University. Approximately 5 × 10^6^ SCC9 cells with OE-NC or OE-PDIA6 stable transfection were suspended in 200 μl of PBS and then subcutaneously injected into the armpit of mice. The tumors were measured to assess tumor formation every week: tumor volume = length × width^2^/2. The mice were euthanized 4 weeks after injection, and the tumor xenografts were harvested and weighed.

### Statistical analysis

Data are expressed as mean ± standard deviation (SD) from 3 independent experiments. SPSS software (version 23.0, SPSS Inc., Chicago, IL, USA) was applied for statistical analyses. Two-side*t* tests and one-way ANOVA with Bonferroni post hoc tests were used to compare differences, such as expression levels, apoptotic rates, absorbance values, migration cell numbers, invasion cell numbers, glucose consumption, lactate production and ATP levels, tumor weights, and tumor volumes, between two and ≥ 3 groups. Kaplan-Meier survival curves with log-rank tests were used to evaluate the relationship between PDIA6 expression levels and OSCC patients’ overall survival. The value of *p* < 0.05 was considered statistically significant.

## Results

### PDIA6 was overexpressed in OSCC tissues

**Fig. 1:**
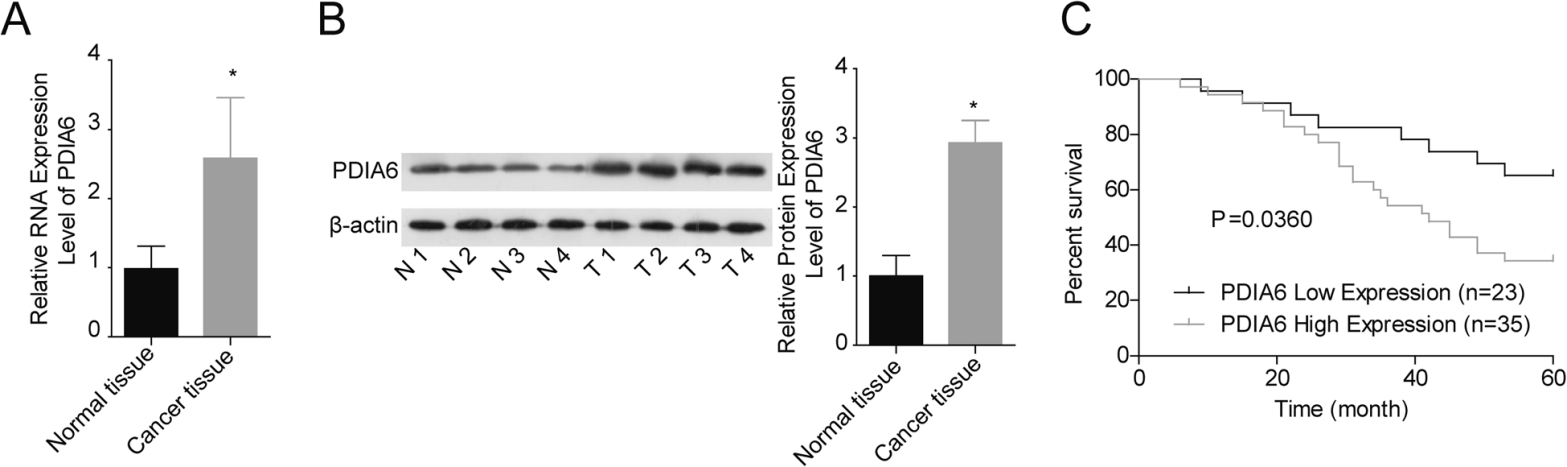
PDIA6 was highly expressed in OSCC tissues and predicted with poor prognosis. **a** The expression levels of PDIA6 mRNA were detected in 58 matched OSCC tissues and the adjacent normal tissues were determined by qPCR. **b** The protein levels of PDIA6 in 4 paired OSCC tissues and the adjacent normal tissues were determined by western blotting assay. **c** Kaplan-Meier analysis of the relationship between PDIA6 mRNA levels and the overall survival of OSCC patients (*p < 0.05)

To explore the role of PDIA6 in the progression of OSCC, we first assessed its expression profile in OSCC tissues. Compared with the adjacent noncancerous tissues, the expression of PDIA6 was significantly elevated in cancer tissues at both mRNA and protein levels, as detected by qPCR (Fig. 1a) and western blotting (Fig. 1b). This result suggested that the high expression of PDIA6 might play a role in the progression of OSCC.

### PDIA6 high expression predicted poor prognosis and malignant clinicopathologic features in OSCC

Then, we evaluated the clinical value of PDIA6 in OSCC. The overall survival for patients of the PDIA6 high expression group was significantly lower than that of the PDIA6 low expression group (Fig. 1c). In addition, the high expression level of PDIA6 was closely associated with higher TNM stage (*p* = 0.006), poorer tissue differentiation (*p* = 0.036), and perineural invasion (*p* = 0.034) in OSCC patients, as shown in Table 2. This result demonstrated that the high expression level of PDIA6 predicted poor prognosis and malignant clinicopathologic features in OSCC.

**Table 2:** Association of PDIA6 expression with clinicopathological features of OSCC patients

Characteristics	Total number (*n* = 58)	PDIA6 expression		*p* value
		Low (*n* = 23)	High (*n* = 35)	
Age (years)				0.074
≤ 60	32	16	16	
> 60	26	7	19	
Gender				0.63
Male	28	12	16	
Female	30	11	19	
Tumor size (cm)				0.217
< 3	27	13	14	
≥ 3	31	10	21	
TNM stage				0.006
I–II	25	15	10	
III–IV	33	8	25	
Tumor differentiation				0.036
Well	28	15	13	
Moderate-poor	30	8	22	
Perineural invasion				0.034
No	33	17	16	
Yes	25	6	19	

### PDIA6 promoted cell growth and inhibited cell apoptosis in OSCC

**Fig. 2:**
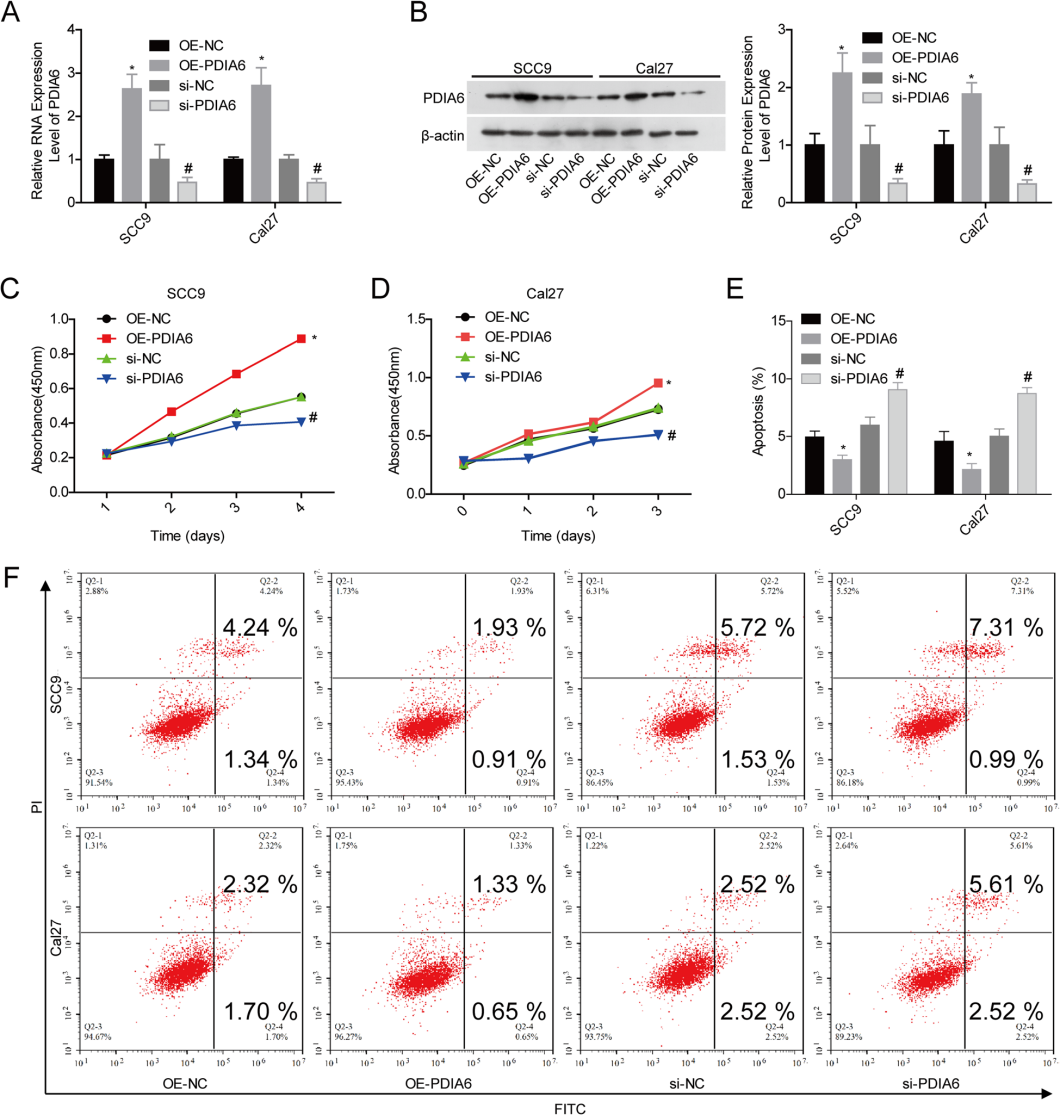
Assessment of PDIA6 role in cell proliferation and apoptosis in SCC9 and Cal27 cells. SCC9 and Cal27 cells were infected/transfected with OE-PDIA6, OE-NC, si-PDIA6, or si-NC, and then collected for detection. **a**, **b** qPCR and western blotting assays were applied to detect the mRNA and protein levels of PDIA6, respectively. **c**, **d** CCK-8 assay was applied for proliferation detection. **e**, **f** Flow cytometry was performed for cell apoptosis detection (*n* = 3, *p < 0.05, OE-PDIA6 group vs. OE-NC group; ^#^*p* < 0.05, si-PDIA6 group vs. si-NC group)

Next, gain- and loss-of-function experiments were carried out to assess PDIA6 role in cell proliferation and apoptosis in OSCC cells. PDIA6 expression was significantly increased following cell infection with OE-PDIA6 in SCC9 and Cal27 cells, while PDIA6 level was decreased when cells were transfected with si-PDIA6 at mRNA and protein levels (Fig. 2a, b). Cell proliferation was significantly enhanced while apoptosis was inhibited when PDIA6 was overexpressed in SCC9 and Cal27 cells, while knockdown of PDIA6 induced an obvious repression in cell proliferation and a significant increase in cell apoptosis rate (Fig. 2c–f). These results demonstrated that PDIA6 promoted OSCC cell growth and inhibited cell apoptosis.

### PDIA6 facilitated OSCC cell migration and invasion

**Fig. 3:**
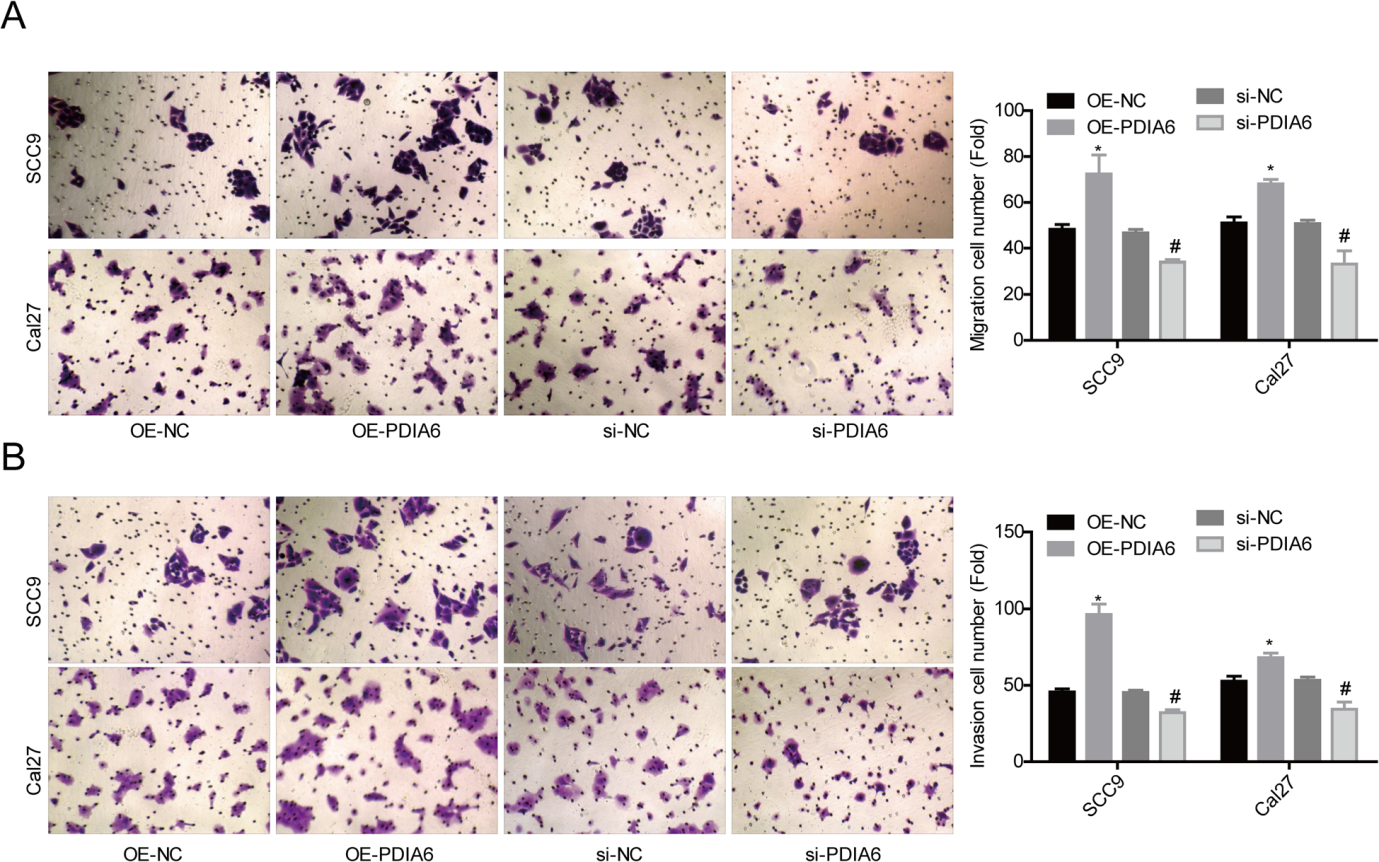
Assessment of PDIA6 role in cell migration and invasion in SCC9 and Cal27 cells. SCC9 and Cal27 cells were infected/transfected with OE-PDIA6, OE-NC, si-PDIA6, or si-NC, and then submitted to transwell chambers assay to detect **a** cell migration and **b** invasion (*n* = 3, *p < 0.05, OE-PDIA6 group vs. OE-NC group; ^#^*p* < 0.05, si-PDIA6 group vs. si-NC group)

In addition, we assessed PDIA6 role in cell migration and invasion in OSCC. Compared with the OE-NC group, cell migration (Fig. 3a) and invasion (Fig. 3b) were all apparently enhanced following PDIA6 overexpression in SCC9 and Cal27 cells, while knockdown of PDIA6 caused opposite results (Fig. 3a, b). These results indicated that PDIA6 served as an inducer in cell migration and invasion in OSCC.

### PDIA6 promoted aerobic glycolysis in OSCC

**Fig. 4:**
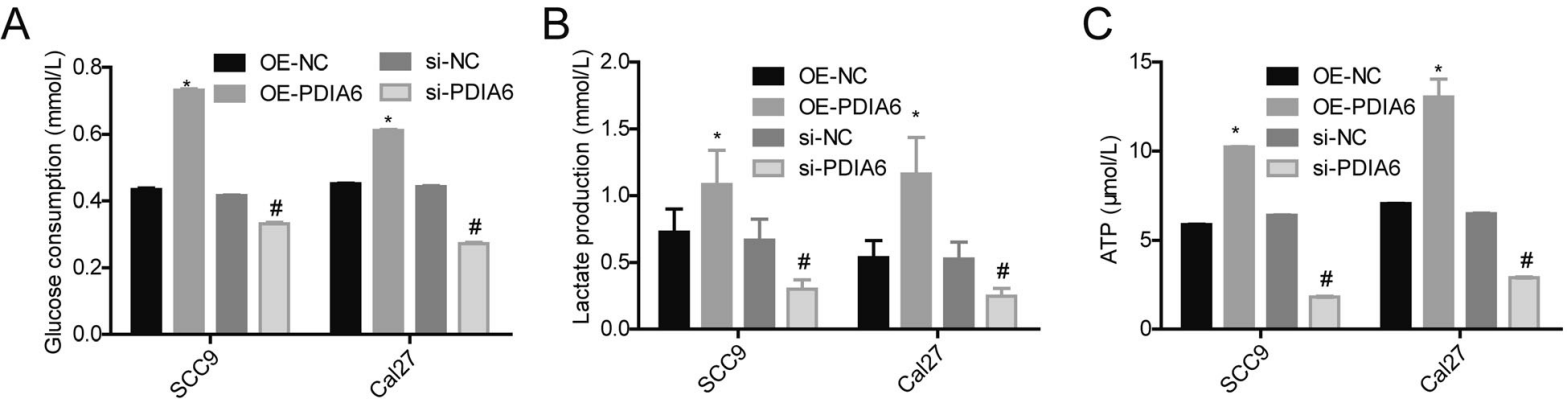
Assessment of PDIA6 role in aerobic glycolysis in SCC9 and Cal27 cells. SCC9 and Cal27 cells infected/transfected with OE-PDIA6, OE-NC, si-PDIA6, or si-NC were collected and submitted to the detection of **a** glucose consumption, **b** lactate production, and **c** ATP level (*n* = 3, *p < 0.05, OE-PDIA6 group vs. OE-NC group; ^#^*p* < 0.05, si-PDIA6 group vs. si-NC group)

Moreover, we evaluated the effect of PDIA6 in the aerobic glycolysis in OSCC cells. Compared with the OE-NC group, the glucose consumption (Fig. 4a), lactate production (Fig. 4b), and ATP level (Fig. 4c) all were significantly increased in the OE-PDIA6 group and were decreased in the si-PDIA6 group. These findings suggested that PDIA6 promoted aerobic glycolysis in OSCC.

### PDIA6 promoted the tumorigenesis of OSCC cells

**Fig. 5:**
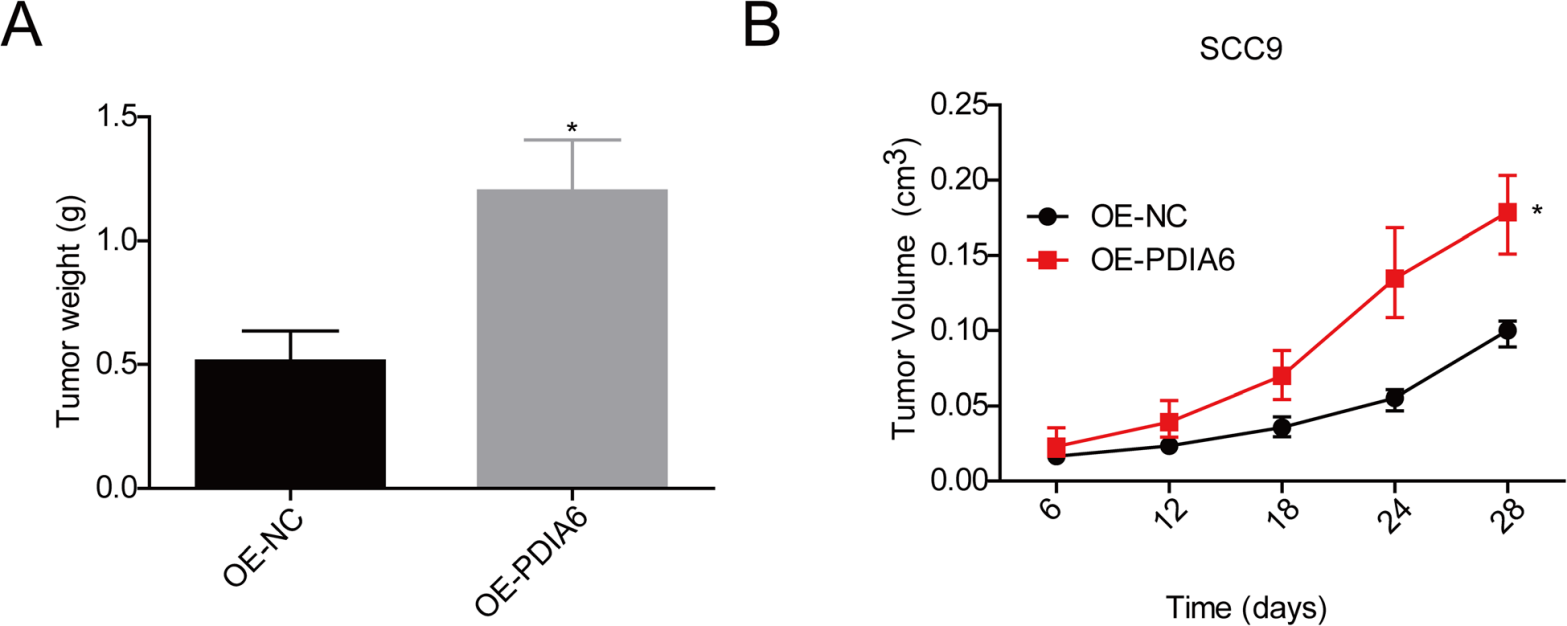
Overexpression of PDIA6 promoted in vivo tumor formation of SCC9 cells. **a** Tumor weights and **b** volume were assessed in mice injected with OE-NC or OE-PDIA6 stably transfected SCC9 cells (*p < 0.05, OE-PDIA6 group vs. OE-NC group)

Finally, we studied PDIA6 role in the in vivo tumor formation ability of SCC9 cells. The result showed that, in comparison with the OE-NC group, the tumor weight and volume were increased when PDIA6 was upregulated in SCC9 cells (Fig. 5a, b), suggesting that PDIA6 enhanced cell tumorigenesis in OSCC.

## Discussion

In the present study, we revealed for the first time that PDIA6 was overexpressed in OSCC tissues and served as an oncogene to promote OSCC growth, migration, invasion, and in vivo tumor formation and inhibit cell apoptosis through enhancing aerobic glycolysis in OSCC. Focusing on aerobic glycolysis, this study provides a potential therapeutic target for OSCC treatment.

Recently, evidence has demonstrated that PDI family members are frequently overexpressed in some cancers with poor prognosis, high incidence of invasion, and chemoresistance [18]. For instance, Ramos et al. [19] suggested that the expression levels of PDIA3 and PDIA6 were increased in primary ductal breast cancer. Bai et al. [15] found that PDIA6 expression was increased in non-small cell lung cancer (NSCLC) tissues, which correlated with poor prognosis; further experiments showed that silencing of PDIA6 inhibited cell growth and promoted cell apoptosis induced by cisplatin, while augment of PDIA6 caused the opposite results. In OSCC, Yuan et al. [20] used the bioinformatics method and immuno-histochemical assays and observed that PDIA3 level was increased in OSCC tissues, which was further verified by the immuno-histochemical assays. Herein, we evaluated the expression profile of PDIA6 in OSCC tissues using qPCR and western blotting assays and found that PDIA6 expression level was increased in OSCC tissues. PDIA6 expression pattern in OSCC was consistent with NSCLC [15], bladder cancer [16], ovarian cancer [21], and hepatocellular carcinoma [17]. Further analysis showed that a higher level of PDIA6 in OSCC patients was closely associated with lower overall survival, higher TNM stage (*p* = 0.006), poorer tissue differentiation (*p* = 0.036), and perineural invasion (*p* = 0.034). Similarly, the PDIA6 expression level was associated with the presence of lymph node metastasis and hormone receptor status in breast cancer [19].

Through the gain- and loss-of-function assays, we found that PDIA6 overexpression significantly promoted the proliferation, invasion, migration, and tumorigenesis of OSCC cells and resulted in a marked decrease in cell apoptosis rate, illustrating that PDIA6 exerted an oncogenic role in OSCC progression. Up to now, PDIA6 has been verified to serve as an oncogene in several kinds of cancers. Inactivation of PDIA6 directly promoted cisplatin-induced cell death in NSCLC [22]. Overexpression of PDIA6 caused an enhancement in cell proliferation accompanied with accelerated cell cycle progression in HeLa cells via activating wnt/β-catenin signaling [23]. Silencing of the PDIA6 gene significantly inhibited bladder cancer cell proliferation, invasion, and in vivo tumor formation and metastasis [16]. Silencing of PDIA6 significantly decreased the tumor volumes to 42.7% in giant cell tumor stromal cells [24]. However, glioblastoma U87MG cell migration and invasion were enhanced significantly after inhibition of PDIA6 [25], suggesting that PDIA6 might play an inhibitory role in cell migration and invasion in glioblastoma. The different cell contents might cause this difference.

It has been recognized that cancer cells present with high glycolytic metabolism even in the presence of oxygen [26, 27]. Glycolysis is beneficial to cancer cells to meet their requirements to maintain rapid growth and migration via providing ATP and metabolic intermediates [28]. Just like other solid cancer cells, OSCC cells get energy to maintain the rapid growth via aerobic glycolysis, resulting in more aggressive behaviors [29, 30]. Wei et al. [31] revealed that the glucose metabolism was significantly increased in premalignant dysplasic oral tissues when compared to the normal oral mucosa tissues, indicating that glycolysis is implicated in the pathogenesis of oral carcinogenesis. In the present study, we explored whether PDIA6 was involved in aerobic glycolysis. The results showed that overexpression of PDIA6 increased glucose consumption, lactate production, and ATP level in OSCC cells. These results suggested that PDIA6 contributed to aerobic glycolysis in OSCC, leading to OSCC progression.

## Conclusion

In summary, the current findings reveal that PDIA6 expression is elevated in OSCC, and predicted poor outcome and advanced clinical process for OSCC patients. Additionally, PDIA6 functions as an oncogene to promote OSCC growth, migration, invasion, and in vivo tumor formation and inhibit cell apoptosis through enhancing aerobic glycolysis.

## Data Availability

All data generated or analyzed during this study are included in this published article.

## Abbreviations

OSCC: Oral squamous cell carcinoma
NSCLC: Non-small cell lung cancer
PDIA6: Protein disulfide isomerase family 6
LDHA: Lactate dehydrogenase A
siRNAs: Small interfering RNAs
qPCR: Real-time quantitative polymerase chain reaction
